# The Screen of a Phage Display Library Identifies a Peptide That Binds to the Surface of *Trypanosoma cruzi* Trypomastigotes and Impairs Their Infection of Mammalian Cells

**DOI:** 10.3389/fmicb.2022.864788

**Published:** 2022-03-10

**Authors:** Jéssica I. de Paula, Eduardo J. Lopes-Torres, Marcelo Jacobs-Lorena, Marcia Cristina Paes, Sung-Jae Cha

**Affiliations:** ^1^Laboratório de Interação Tripanossomatídeos e Vetores – Departamento de Bioquímica, IBRAG – UERJ, Rio de Janeiro, Brazil; ^2^Laboratório de Helmintologia Romero Lascasas Porto, Faculdade de Ciências Médicas, Universidade do Estado do Rio de Janeiro, Rio de Janeiro, Brazil; ^3^Johns Hopkins Bloomberg School of Public Health, Department of Molecular Microbiology and Immunology and Malaria Research Institute, Baltimore, MD, United States; ^4^Instituto Nacional de Ciência e Tecnologia - Entomologia Molecular (INCT-EM), Rio de Janeiro, Brazil

**Keywords:** *Trypanosoma cruzi*, phage display, peptide library, infection, trypomastigote

## Abstract

**Background:**

Chagas is a neglected tropical disease caused by the protozoan parasite *Trypanosoma cruzi*. On the order of seven million people are infected worldwide and current therapies are limited, highlighting the urgent need for new interventions. *T. cruzi* trypomastigotes can infect a variety of mammalian cells, recognition and adhesion to the host cell being critical for parasite entry. This study focuses on trypomastigote surface ligands involved in cell invasion.

**Methods:**

Three selection rounds of a phage peptide display library for isolation of phages that bind to trypomastigotes, resulted in the identification of the N3 dodecapeptide. N3 peptide binding to *T. cruzi* developmental forms (trypomastigotes, amastigotes and epimastigotes) was evaluated by flow cytometry and immunofluorescence assays. Parasite invasion of Vero cells was assessed by flow cytometry and immunofluorescence assays.

**Results:**

Phage display screening identified the N3 peptide that binds preferentially to the surface of the trypomastigote and amastigote infective forms as opposed to non-infective epimastigotes. Importantly, the N3 peptide, but not a control scrambled peptide, inhibits trypomastigote invasion of Vero cells by 50%.

**Conclusion:**

The N3 peptide specifically binds to *T. cruzi*, and by doing so, inhibits Vero cell infection. Follow-up studies will identify the molecule on the parasite surface to which the N3 peptide binds. This putative *T. cruzi* ligand may advance chemotherapy design and vaccine development.

## Introduction

The protozoan parasite *T. cruzi* is the causative agent of Chagas disease, also known as American trypanosomiasis. Currently, about 6 to 7 million people are infected worldwide, most cases occurring in Latin America (World Health Organization [WHO], 2021). Chagas disease is endemic in Latin America but because of migration, the disease has spread beyond endemic regions to become a worldwide health problem (Schmunis and Yadon, 2010; Requena-Méndez et al., 2015; Nguyen and Waseem, 2021).

The *T. cruzi* life cycle involves two hosts, mammals and the insect vector. There are four main developmental stages: the replicative epimastigote and amastigote forms, and the non-replicative infective metacyclic and bloodstream trypomastigote forms (Rassi et al., 2010). Amastigotes can infect vertebrate cells (Fernandes et al., 2013). Trypomastigotes can invade most nucleated cells of the vertebrate host and are crucial to establish intracellular infection (Burleigh and Andrews, 1995). The infection process is complex and involves a large repertoire of molecules on the parasite surface, including *trans*-sialidases and glycoproteins such as gp82 and gp85 (De Pablos and Osuna, 2012; Cortez et al., 2014; San Francisco et al., 2017; Campetella et al., 2020). Identification of parasite ligand(s) required for cell infection may lead to the development of new drugs and a vaccine against Chagas disease.

Despite the unsuccessful attempts of new treatments, such as posaconazole and the prodrug of ravuconazole, the treatment of Chagas disease is still restricted to two drugs, benznidazole and nifurtimox, both having safety and efficacy profiles that are far from ideal (Pérez-Molina and Molina, 2018). The discovery of new treatment alternatives is urgently needed. Molecules on the surface of the parasite can serve as targets for the development of new drugs or vaccines. In this context, the use of phage peptide display screening is a simple and efficient approach for identifying peptides that target parasite infection, which in turn may lead to the development of new drugs and vaccines (Rahbarnia et al., 2017; Bao et al., 2019).

Here we report on the screening of a phage display library for peptides that bind to the surface of *T. cruzi* trypomastigotes. This led to the identification of the N3 peptide that selectively binds to *T. cruzi* trypomastigotes and by doing so, inhibits host cell infection.

## Materials and Methods

### *Trypanosoma cruzi* and Mammalian Host Cell Culture

*Trypanosoma cruzi* epimastigotes (strain Y) were grown in Brain Heart infusion (BHI) with 10% fetal calf serum (FCS) in the presence of hemin (30 μM) at 28°C for 7 day.

Tissue-derived *T. cruzi* trypomastigotes (strain Y) were obtained from the supernatant of infected Vero cells 6 days after infection. The infected Vero cells were cultured in Dulbecco’s modified Eagle’s medium (DMEM) supplemented with penicillin (100 units/ml), streptomycin (100 mg/ml), sodium bicarbonate plus 5% FCS at 37°C in a humidified atmosphere with 5% CO_2_. Extracellular amastigotes were obtained from the 15th day post infection without change of medium from the supernatant of infected Vero cells.

Vero cells (ATCC^®^ CCL-81™) were cultured in DMEM plus 10% FCS at 37°C in a humidified atmosphere with 5% CO_2_.

### Phage Selection

**FIGURE 1:**
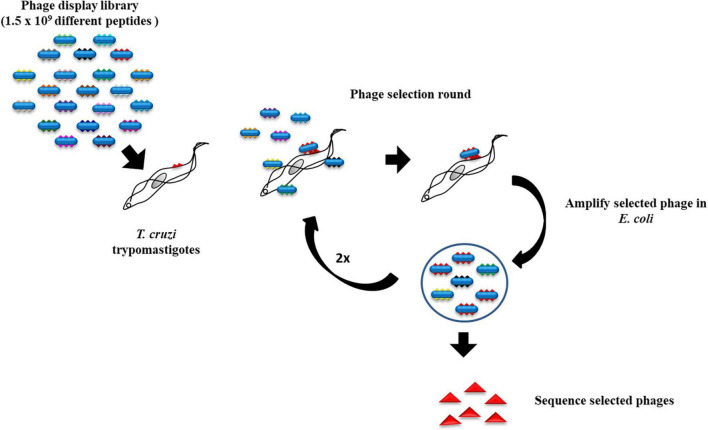
Strategy for the screening of a phage display library. *Trypanosoma cruzi* trypomastigotes were incubated with a phage display library followed by washing to remove the loosely bound phages. After three rounds of selection, phages that bound to the trypomastigotes were plated and the DNA from random colonies coding for the displayed peptides was sequenced.

A phage library displaying 12-amino acid peptides fused to the N terminal of the pVIII coat protein on the surface of M13 filamentous phage f88.4 (Cha et al., 2015) was used. The composition of the peptides is random, except for fixed cysteines at positions 2 and 11, which form a disulfide bond resulting in an eight-amino acid loop. The phage library has a complexity of 1.5 × 10^9^ different peptides. The recombinant phages were amplified in K91 *Escherichia coli* grown in LB media plus 10 μg/ml tetracycline. The selection was initiated by incubating 10^11^ phages with 10^9^ trypomastigotes for 30 min on ice in DMEM without FBS. The trypomastigotes were then washed six times with PBS to remove unbound and weakly bound phages from the parasites. Bound phages were amplified in the host *E. coli* and precipitated from the culture supernatant by adding an equal volume of 16% polyethylene glycol 8000 (PEG8000, Fisher Scientific). The amplified phages were tittered and used for a new selection as described previously (Cha et al., 2015) performed to enrich for strong binders (Figure 1). At the end of three rounds of selections, 41 random phage colonies were picked for sequencing of the DNA coding for the peptide inserts (Table 1).

**TABLE 1:** Sequence analysis of 41 random phage clones after the third round of selection.

Phage	Frequency	Nucleotide sequence	Predicted peptide sequence
N1	7/41 (17%)	CAGTGTATTGATTTTAAGGTGTATACTTGGTGTCCG	QCIDFKVYTWCP
N2	1/41 (2.5%)	GGGTGTGAGGCTTGGTGGTGGTGGAAGCCTTGTAAT	GCEAWWWWKPCN
N3	12/41 (29%)	CAGTGTATTCAGTTTCCTCAGTTTCTGTGGTGTCCT	QCIQFPQFLWCP
N4	1/41 (2.5%)	ATTTGTCATAGGGATATGTGGTAGTATTCGTGTATG	ICHRDMW*YSCM
N5	1/41 (2.5%)	GCATGTGGTTCACTGGTGAGTATAATGCGCTGTATC	ACGSLVSIMRCI
N6	1/41 (2.5%)	GAGTGTGAGTGGGTTTAGCATCTGAGTTTTTGTCCT	ECEWV*HLSFCP
N7	1/41 (2.5%)	CATTGTGGGGAGGTGGATAGTCATGCTGAGTGTCCG	HCGEVDSHAECP
N8	1/41 (2.5%)	CCTTGTACTGGGAGGTAGAATGTGACTCAGTGTCTG	PCTGR*NVTQCL
N9	1/41 (2.5%)	GGGTGTCCGATGCTAAGCAGAAGCTTACTTTGTCTG	GCPMLSRSLLCL
N10	1/41 (2.5%)	CTTTGTCATTAGTCTTCTTGTGCTGCGTTGTGTGAG	LCH*SSCAALCE
N11	1/41 (2.5%)	ATGGGTAAGCCTTCTTATGCGGGTACTGCTTGTACT	MGKPSYAGTACT
N12	1/41 (2.5%)	CAGTGTAATCAGTGGCCTCAGTTTCATCGGTGTCAG	QCNQWPQFHRCQ
N13	1/41 (2.5%)	GATTGTAGAGGGGCGTGTTGTTCGTATGATTGTCCT	DCRGACCSYDCP
N14	1/41 (2.5%)	ATGAGTCAGGCGCCTAATCCTAAGCGGTGTTGTATG	MSQAPNPKRCCM
N15	1/41 (2.5%)	AGGTGTACGAAGCGTGTTTTTCTGGTTAATTGTGGG	RCTKRVFLVNCG
N16	1/41 (2.5%)	TTGTGTACGCTGCCTTCTATTACTAATACTTGTAAT	LCTLPSITNTCN
N17	2/41 (5.0%)	ATGGGTCCGCATCCTTCTGATGTGCCTAAGTGTGCG	MGPHPSDVPKCA
N18	1/41 (2.5%)	CAGTGTCCGAAGCGCGAGTCTGCCGTGGTCTGTTTG	QCPKRESAVVCL
N19	1/41 (2.5%)	GCTTGTAGTTTGCTTACTTATAAAGGCTAATGTGTA	ACSLLTYKG*CV
N20	1/41 (2.5%)	GATTGTAGGAAGGTTTGGATTCGTAGTAAGTGTGTG	DCRKVWIRSKCV
N21	1/41 (2.5%)	GATTGTAGGAAGGTTTGGATTCGTAGTAAGTGTGTG	GCLEEGSWFFCK
N22	1/41 (2.5%)	CATTGTCAGACTCTTTTGGCTGTTGATGGTTGTACT	HCQTLLAVDGCT
N23	1/41 (2.5%)	ATGAGTCAGGCGCCTAATCCTAAGCGGTGTTGTATG	QCKKAWRITPCL

Times/chamber for statistical analysis. *Indicates a stop codon.

### Peptide Synthesis and 3D Conformation

**FIGURE 2:**
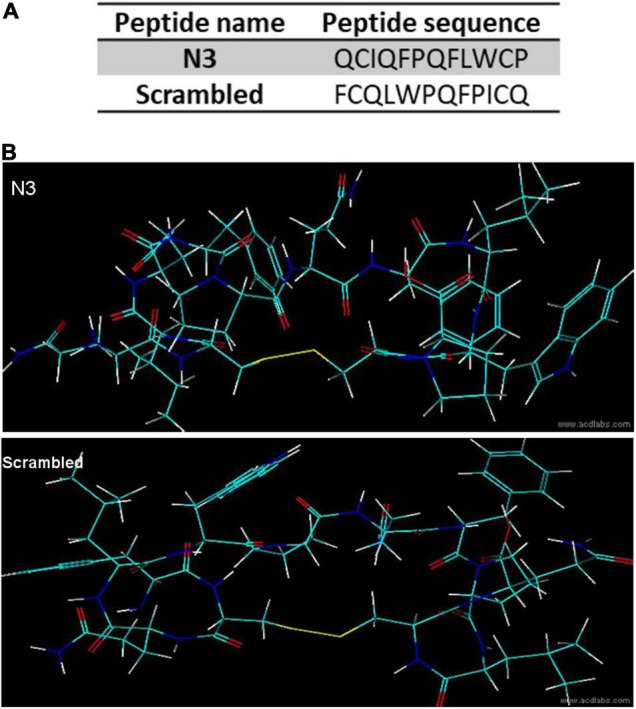
Peptide sequence and predicted structure. **(A)** Peptide primary sequences. **(B)** 3D predicted structures.

Peptide 2.0 Inc., synthesized an N-terminal biotinylated N3 peptide, circularized *via* a disulfide bridge between the two cysteines. A control scrambled peptide, having the same amino acid composition but different sequence, was also synthesized (Figure 2A).

Peptide three-dimensional structures (Figure 2B) were predicted with the ACD/Labs 2018.2.1 (Advanced Chemistry Development, Inc.) ACD/ChemSketch and ACD/3D view softwares.

### Binding Assays

#### Flow Cytometry for N3 Peptide Binding to *Trypanosoma cruzi*

*Trypanosoma cruzi* developmental forms, trypomastigotes, amastigotes and epimastigotes, or Vero cells were fixed with 4% paraformaldehyde for 1 h and blocked with 4% bovine serum albumin for 1 h. Next, parasites or Vero cells were incubated overnight with 0.15 mg/ml of biotinylated peptide (N3 or scrambled). After triple washes with PBS, bound peptides were visualized with Alexa fluor 594-conjugated streptavidin (excitation 591 nm and emission 620 nm). Fluorescence intensity was determined using a Gallios Flow Cytometer (Beckman Coulter).

#### Fluorescence Assays for N3 Peptide Binding to *Trypanosoma cruzi* Trypomastigotes

Peptide binding assays to trypomastigotes followed the same procedures as for the flow cytometry assays. Parasites were attached to coverslips precoated with 0.01% poly-L-lysine (Sigma-Aldrich), mounted in ProLong^®^ Gold antifade with 4′,6-diamidino-2-phenylindole (DAPI) reagent (Invitrogen) and analyzed with an Olympus BX51 fluorescence microscope.

### Host Cell Invasion

Trypomastigotes were incubated with 0.15 mg/ml peptide (N3 or scrambled) for 15 min on ice, and then fluorescently labeled with succinimidyl ester of carboxyfluorescein (CFSE) according to the manufacturer’s instructions (Thermo Fisher Scientific; catalog number C34554). After labeling, parasites were used to infect Vero cells (10:1, parasites: Vero) for 3 h and followed by flow cytometry and immunofluorescence assays.

#### Flow Cytometry

Infected Vero cells in a 6-well plate were trypsinized, washed with PBS and fixed in 1% paraformaldehyde. Vero cell intracellular fluorescence was quantified with Gallios (Beckman Coulter) and analyzed with the Kaluza 1.2 software (Beckman Coulter). The invasion intensity was determined by CFSE fluorescence intensity. The cytometer was adjusted for zero fluorescence with non-infected Vero cells (negative control).

#### Immunofluorescence Assay

Vero cells were plated and infected in an 8-well chamber slide (Lab-Tek II). After 3 h of interaction, free parasites were removed, and cells were fixed in 1% paraformaldehyde followed by visualization and quantification of intracellular parasites with a fluorescence microscope. Invasion intensity was determined by measuring the average number of parasites per 100 cells.

### Statistical Analysis

Data were analyzed using the GraphPad Prism program. For all results, the One-way ANOVA post Tukey or Dunnet test was applied.

## Results

### Screening for Peptides With High Affinity to Trypomastigotes’ Surface

Trypomastigotes, the infective *T. cruzi* form, can invade a large variety of cell types of its vertebrate host. We screened a phage peptide display library to identify peptides that bind with high affinity to the surface of trypomastigotes (Figure 1). The estimated complexity of the library is 1.5 × 10^9^ different 12-amino acid peptides. To select peptides that have high affinity for trypomastigotes, 10^11^ library phages were incubated with 10^9^ trypomastigotes (∼100 phages per parasite) and weakly bound phages were removed by washing. Bound phages were amplified by adding host *E. coli* cells and used for the next round of selection. After the third round, 41 random phage colonies from two independent experiments were picked for sequencing of the DNA encoding the displayed peptide. About 30% of the phages (12/41)–termed N3–displayed the same peptide, and 17% (7/41)–termed N1–displayed the second most frequent peptide. The remaining phages displayed peptides represented only once or twice (Table 1). Considering the high complexity of the initial phage library, the high proportion of phages displaying the same peptide suggests that they specifically bind to trypomastigotes. For further experiments, we had two peptides synthesized: the N3 peptide and a scrambled peptide with the same amino acid composition but different sequence, to be used as a specificity control (Figure 2).

### The N3 Peptide Binds Specifically to Infective Parasite Forms

**FIGURE 3:**
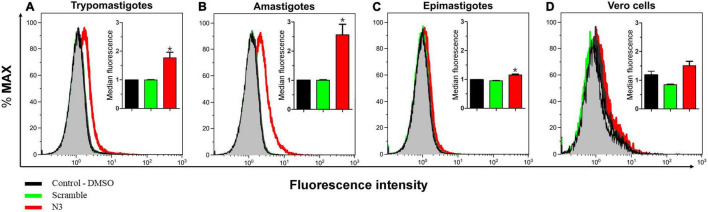
Assay of N3 peptide binding to different *Trypanosoma cruzi* forms and host cells using flow cytometry. Trypomastigotes **(A)** Amastigotes **(B)** Epimastigotes **(C)**, and Vero cells **(D)** were fixed with paraformaldehyde and incubated overnight with biotinylated N3 or scrambled peptides. Bound peptides were detected with fluorescently labeled streptavidin and fluorescence intensity was quantified by flow cytometry. The insert shows median fluorescence from three independent experiments. *P < 0.05 relative to control by one-way ANOVA pos-test Tukey.

**FIGURE 4:**
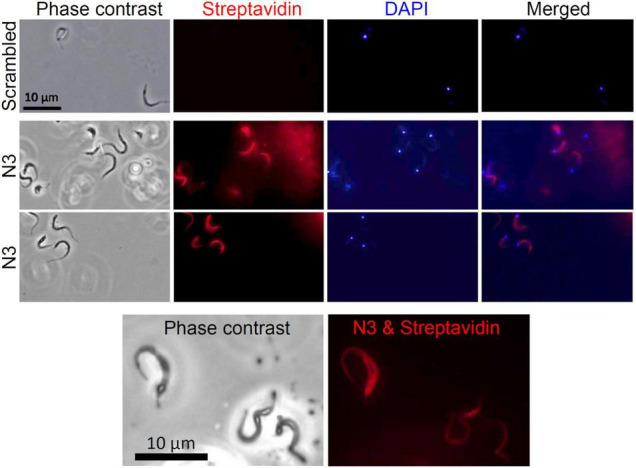
Assay of N3 peptide binding to trypomastigotes using fluorescence microscopy. Trypomastigotes were fixed with paraformaldehyde and incubated overnight with biotinylated N3 or scrambled peptides. Bound peptides were detected with fluorescently labeled streptavidin and cells were viewed with fluorescence microscopy. No fluorescence was detected when trypomastigotes were incubated with the biotinylated scrambled peptide.

To test the specificity of the N3 peptide interaction with *T. cruzi*, we incubated biotinylated N3 and scrambled peptides with trypomastigotes, amastigotes, epimastigotes and with host Vero cells, and assayed for peptide binding using flow cytometry (Figure 3). The median fluorescence of the N3 peptide binding to trypomastigotes and amastigotes forms that can infect mammalian host cells is 1.7 and 2.5-fold higher than the control, respectively. N3 peptide binding to epimastigotes is 1.2-fold higher than control, and no significant binding to the Vero cells was detected. The control scrambled peptide bound neither to parasites nor to host cells. These results were confirmed by fluorescence microscopy. As shown in Figure 4, the N3 peptide, but not its scrambled counterpart, binds to trypomastigotes.

#### The N3 Peptide Inhibits Trypomastigote Infection of Vero Cells

**FIGURE 5:**
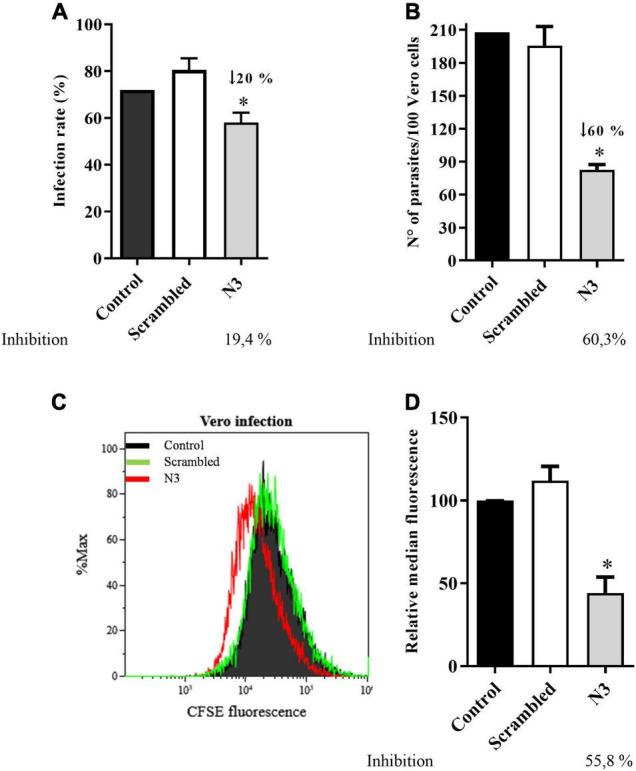
*Trypanosoma cruzi* infection of Vero cell is impaired by the N3 peptide. Trypomastigotes were fluorescently labeled by incubation with CFSE, followed by incubation with N3 or scrambled peptides and then used to infect Vero cells. Percent cells infected **(A)** and the number of parasites inside Vero cells **(B)** was quantified by counting fluorescent parasites by fluorescence microscopy. Data are representative of three independent experiments. Infection was also assayed by flow cytometry. A representative profile is shown in **(C)** and mean fluorescence combined from three independent experiments is shown in **(D)**. *p < 0.05 relative to control by one-way Anova pos-test Tukey.

The specific binding of the N3 peptide to the infective *T. cruzi* forms raises two possible interpretations: (1) N3 binds to a *T. cruzi* surface molecule involved in host cell infection or (2) N3 binds to a molecule unrelated to infection. To distinguish between the two possibilities, trypomastigotes were fluorescently labeled by incubation with CFSE (carboxyfluorescein diacetate succinimidyl ester) and incubated with peptide (N3 or scrambled) before Vero cell interaction assays. Free parasites were then removed by washing and infection intensity was analyzed by fluorescence microscopy and flow cytometry. Microscopic counting found that N3 peptide treatment reduced infection rate (% Vero cells infected) by 20% compared to no peptide control (Figure 5A), and infection intensity (number of parasites per host cell) was reduced by 60% (Figure 5B). The scrambled peptide had no significant effect. These results were confirmed when evaluated by flow cytometry. Figures 5C,D shows that N3 peptide treatment decreases fluorescence, indicating a reduction of parasite infection. This was confirmed by comparison of median fluorescence intensities: N3 peptide treatment reduced fluorescence intensity by 55% compared to the no-peptide control.

## Discussion

Peptides identified with the phage display approach have been used clinically to treat diseases. For instance, Ecallantide is a serine protease inhibitor that has been used to treat hereditary angioedema (Markland et al., 1996; Ladner et al., 2007; Farkas and Varga, 2011; MacGinnitie et al., 2012). Another example is Romiplostim (Nplate®), a thrombopoietin receptor agonist used to treat thrombocytopenia (Cwirla et al., 1997; Keating, 2012; Tarantino et al., 2019). The phage display approach led to the identification of a peptide that structurally mimics glyceraldehyde-3-phosphate dehydrogenase (GAPDH) on the surface of *Plasmodium* sporozoites; this peptide became a new vaccine candidate for targeting malaria liver invasion (Cha et al., 2016, 2018). The phage display approach was used to search for mimotopes of the *Trypanosoma brucei* variant surface glycoprotein (VSG). These mimotopes are important for diagnosing the disease using an antibody detection test (van Nieuwenhove et al., 2011, 2012). In *Leishmania*, the technique was used for vaccine development. The serum of patients with cutaneous (*L. amazonensis* infection) or visceral (*L. infantum* infection) leishmaniasis was used for biopanning. The selected peptides were immunogenic, inducing specific T helper 1 (Th-1) response (Ramos et al., 2017; Carvalho et al., 2019). Phage display is also being used to diagnose canine leishmaniasis and as a marker for human visceral leishmaniasis (Costa et al., 2019; Machado et al., 2019).

In *T. cruzi*, the phage display contributed to elucidate the interaction of glycoproteins from the *trans-*sialidase/gp85 family with mammalian host cells. In this context, the use of phages expressing FLY (a *trans-*sialidase/gp85 motif peptide) demonstrated the participation of the enzyme in the interaction with endothelial cells. In addition, an important new motif of *trans-*sialidase/gp85 (TS9) and the receptor on the host cell that binds to gp85, the prokineticin-2 receptor (PKR2), has been identified (Tonelli et al., 2010; Khusal et al., 2015; Teixeira et al., 2015). Recently, phage display has been used to study immune response in order to find new epitopes and antigens or to understanding pathological manifestation in Chagas Disease (Niborski et al., 2021; Teixeira et al., 2021). Furthermore, phage display screening led to the identification of a peptide (named EPI18) that binds to the surface of the non-infective epimastigote form (Sáenz-Garcia et al., 2020).

The trypomastigote is the classical infectious form of *T. cruzi*, although invasion of vertebrate host cells by amastigotes has also been described. *T. cruzi* uses several mechanisms and surface molecules to enter the host cell (Caradonna and Burleigh, 2011; Nagajyothi et al., 2011; Leiria Campo et al., 2016). Entry of the host cell is essential to complete the parasite’s life cycle and infection spread, thus this is a promising target for the development of new therapeutic strategies (Walker et al., 2013). In this work, we used the same phage library that was used to identify a GAPDH ligand molecule on the surface of the invasive *Plasmodium* sporozoite (Cha et al., 2016, 2018). Here we showed that the N3 peptide binds to the *T. cruzi* invasive forms (Lima et al., 2010; Salassa and Romano, 2018) and importantly, by doing so, inhibits target cell infection. Our results also suggest that N3 is relevant for other stages of infection besides the interaction itself, as the load significantly decreases within the infected cell. As N3 did not bind to the non-infective forms, the possibility arises that the infective trypomastigotes and amastigotes display on their surface a common ligand that is recognized by N3. Investigation of this possibility is under way.

## Conclusion

A phage display screen led to the identification of the N3 peptide that binds to the surface of trypomastigotes and inhibits host mammalian cell infection. Future identification of the target molecule may lead to the development of a new vaccine antigen and therapeutic target.

## Data Availability Statement

The original contributions presented in the study are included in the article/supplementary material, further inquiries can be directed to the corresponding authors.

## Author Contributions

JP, MP, S-JC, and MJ-L contributed to the conception and design of the study. JP and S-JC performed the experiments. JP, MP, EL-T, and S-JC contributed to analyses of data. JP wrote first version of manuscript. MP, S-JC, and MJ-L provided input for manuscript revisions. All authors contributed to the article and approved the submitted version.

## Conflict of Interest

The authors declare that the research was conducted in the absence of any commercial or financial relationships that could be construed as a potential conflict of interest.

## Publisher’s Note

All claims expressed in this article are solely those of the authors and do not necessarily represent those of their affiliated organizations, or those of the publisher, the editors and the reviewers. Any product that may be evaluated in this article, or claim that may be made by its manufacturer, is not guaranteed or endorsed by the publisher.
